# Supplementary material for: Development of a novel non-invasive biomarker panel for hepatic fibrosis in MASLD
Source: Nat Commun. 2024 May 29;15:4564. doi: 10.1038/s41467-024-48956-0 (PMC11137090; doi:10.1038/s41467-024-48956-0)
Supplement: Supplementary file 1 — Supplementary Information [file 41467_2024_48956_MOESM1_ESM.pdf]

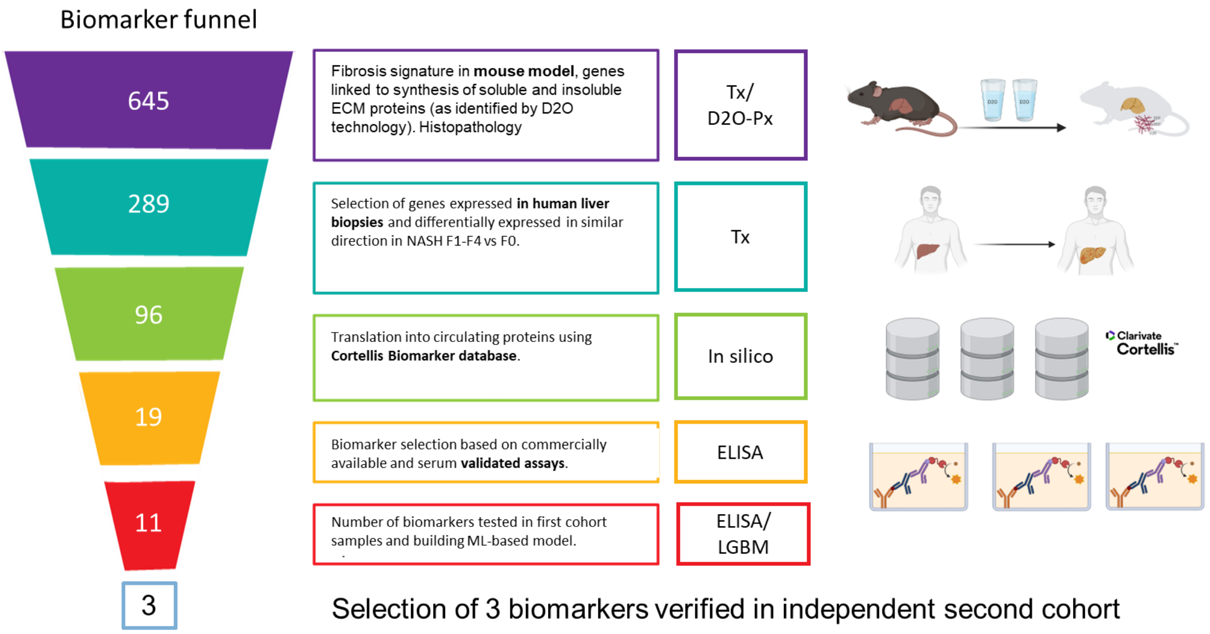

Supplemental Figure 1: A graphical overview of the steps taken in the biomarker identification approach. Created with BioRender.com, released under a Creative Commons Attribution-NonCommercial-NoDerivs 4.0 International license.

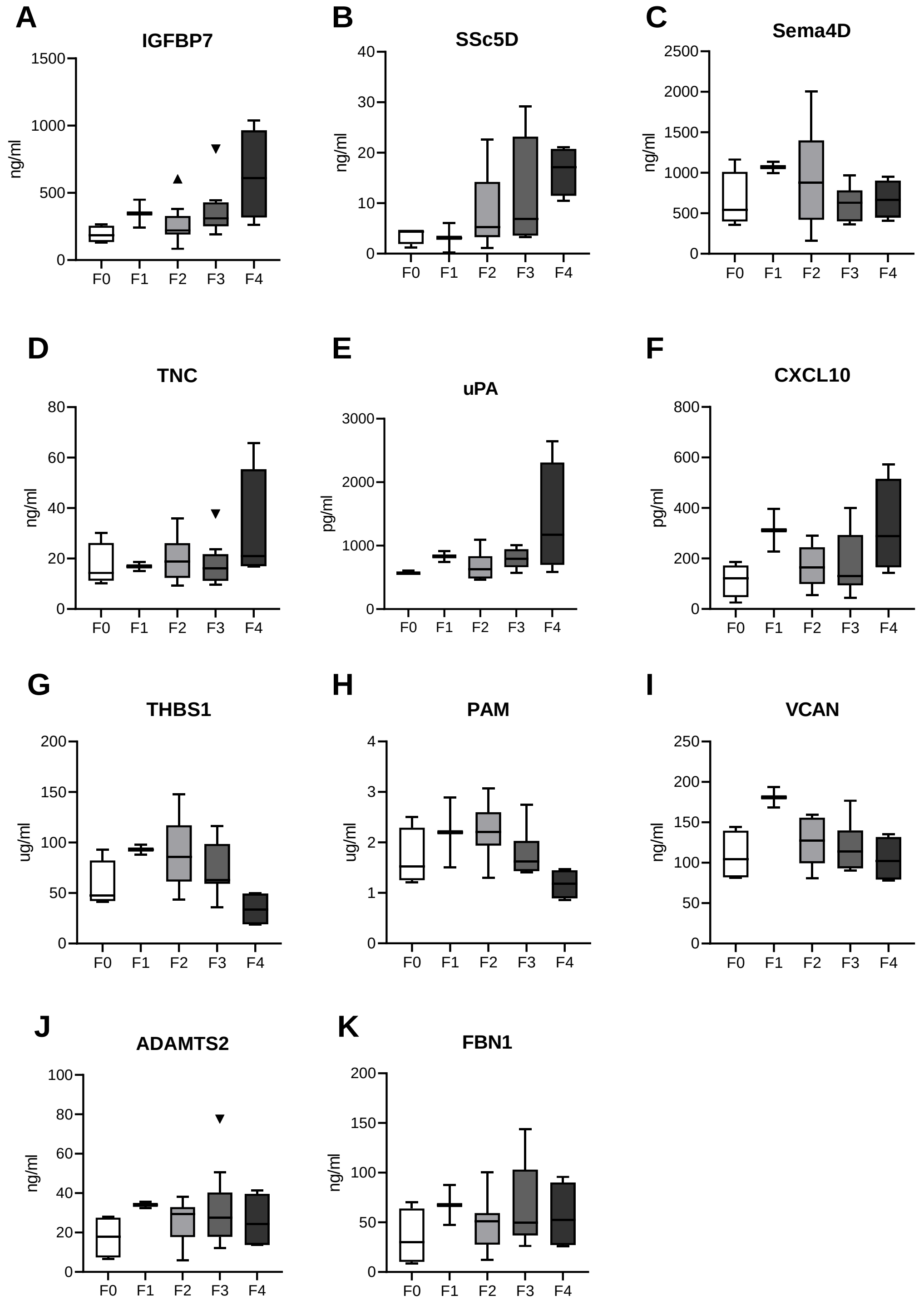

Supplemental Figure 2: Serum levels of a) IGFBP7, b) SSc5D, c) SEMA4D, d) TNC, e) uPA, f) CXCL10, g) THBS1, h) PAM, i) VCAN, j) ADAMTS2, and k) FBN1 in samples from patients in the translation cohort with biopsy-proven stages of fibrosis. F0, n=4; F1, n=2; F2, n=10; F3, n=9; F4, n=4). Values are shown in box and whisker plots, data are median (horizontal line), interquartile range (boxes) and 25th-75th percentile (error bars). The triangles indicate outliers.

A

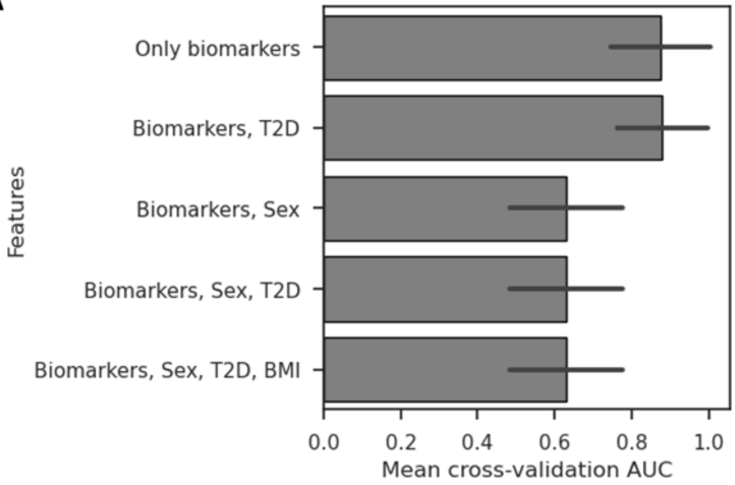

B

Features	Mean AUC	SD AUC
Biomarkers, T2D	0.880556	0.116150
Only biomarkers	0.876389	0.129813
Biomarkers, Sex	0.631250	0.146812
Biomarkers, Sex, T2D	0.631250	0.146812
Biomarkers, Sex, T2D, BMI	0.631250	0.146812

C

Gender	subgroup	Total	Accuracy
female	0/1	30	0.63
female	2	15	0.53
female	3/4	29	0.72
male	0/1	36	0.75
male	2	15	0.27
male	3/4	31	0.61

Supplemental Figure 3: A) Mean and standard deviation of AUROC curves (20-fold cross-validation) generated by the LightGBM model by recursively adding clinical variables. B) table with data to show the effect of the biomarker performance in predicting fibrosis in MASLD in the testing cohort. C) Table with data to show LGBM model accuracy per gender in the independent validation cohort.

Values are expressed as mean ± SD

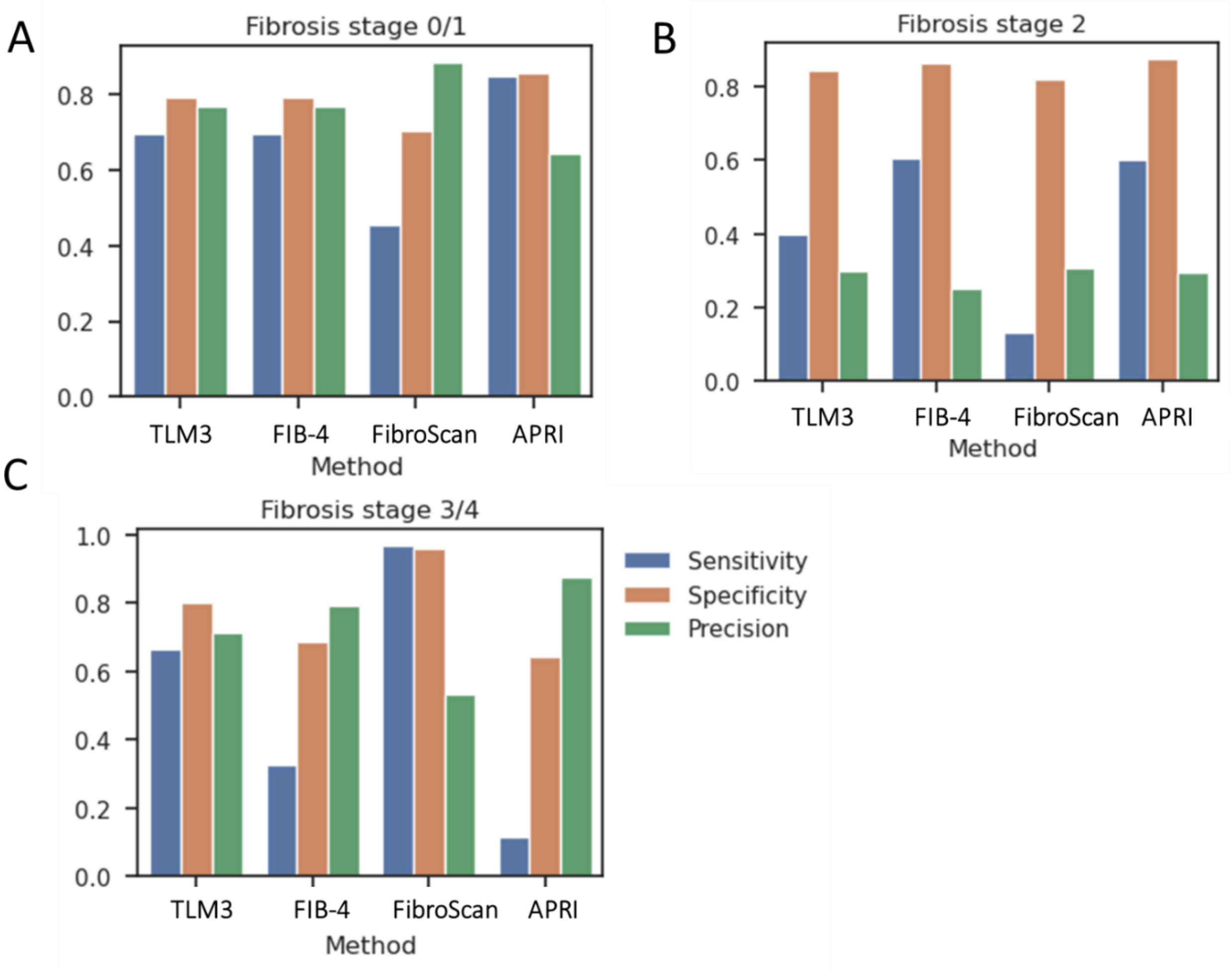

Supplemental Figure 4: Overview of model performances of different NITs in Sensitivity, Specificity, and Precision of A) Fibrosis stages F0/F1, B) Fibrosis stage F2, and C) Fibrosis stages F3/F4.

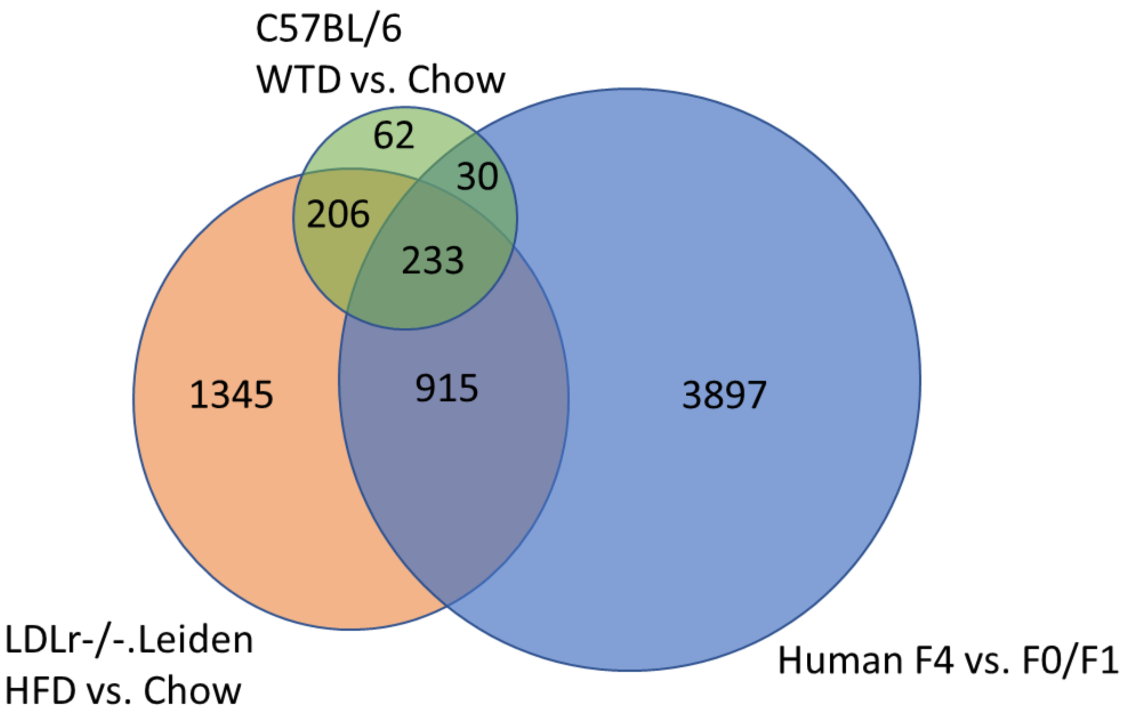

Supplemental Figure 5: Venn diagram showing the overlap of DEGs in the Western-type diet-treated C57BL/6 mice and High fat diet-treated LDLr-/-.Leiden mice as compared to respective chow fed controls. The DEGs of the mouse studies were compared to DEGs (all Padj<0.001) in human liver (comparison of biopsy confirmed F4 vs. F0/1 livers from MASLD patients).

	Mouse COL1A1	Human COL1A1
IGFBP7	***0.89***	***0.57***
SSC5D	0.99	***0.80***
SEMA4D	***0.87***	***0.58***
TNC	0.96	***0.21***
PLAU	0.96	***0.41***
CXCL10	***0.78***	***0.28***
THBS1	***0.82***	***0.49***
PAM	***0.86***	***0.22***
VCAN	0.96	***0.42***
ADAMTS2	***0.86***	***0.68***
FBN1	0.95	***0.56***

Supplemental Table 1: Correlation coefficient of genes in the mouse study (column 1) related to the expression of COL1A1. Correlation coefficients of candidate biomarker genes with COL1A1 expression in human MASLD patients (column 2). Genes in bold and italic would not have been selected by correlation analysis using COL1A1 mRNA expression.

A)

**FIB4 confusion matrix**		Predicted class				Perfomance metrics				
		F0/1	F2	F3/4				Sensitivity	Specificity	Precision
True class	F0/1	59	21	4			F0/1	0.70	0.83	0.52
	F2	32	29	7			F2	0.43	0.75	0.28
	F3/4	23	55	31			F3/4	0.28	0.64	0.74

B)

**TLM3 confusion matrix**		Predicted class				Perfomance metrics				
		F0/1	F2	F3/4				Sensitivity	Specificity	Precision
True class	F0/1	51	16	7			F0/1	0.69	0.82	0.76
	F2	8	19	11			F2	0.50	0.87	0.36
	F3/4	8	18	56			F3/4	0.68	0.78	0.76

Supplemental Table 2: Confusion matrices and performance metrics of FIB4 (A) and TLM3 (B) and of all samples used in the study. The left part of the table presents the confusion matrix, and the right part the corresponding performance matrices (sensitivity, specificity and precision).
