# Supplementary material for: Development of a novel non-invasive biomarker panel for hepatic fibrosis in MASLD
Source: Nat Commun. 2024 May 29;15:4564. doi: 10.1038/s41467-024-48956-0 (PMC11137090; doi:10.1038/s41467-024-48956-0)
Supplement: Supplementary file 3 — Description of Additional Supplementary Files [file 41467_2024_48956_MOESM3_ESM.pdf]

Description of Additional Supplementary Files

Supplementary Data 1: List of differentially expressed genes and their correlation the fractional synthesis rate of at least one of the selected fibrillar collagens. A two-sided Student's t-test was used to test the statistical significance.

Supplementary Data 2: Overview of the Fibrosis stages in patients that were included in the translation cohort.
